# TLR3 Signaling in Macrophages Is Indispensable for the Protective Immunity of Invariant Natural Killer T Cells against Enterovirus 71 Infection

**DOI:** 10.1371/journal.ppat.1004613

**Published:** 2015-01-23

**Authors:** Kai Zhu, Juhao Yang, Kaiming Luo, Chunhui Yang, Na Zhang, Ruifeng Xu, Jianxia Chen, Mingfei Jin, Bin Xu, Nining Guo, Jianrong Wang, Zuolong Chen, Ying Cui, Hui Zhao, Yan Wang, Chaoyang Deng, Li Bai, Baoxue Ge, Cheng-Feng Qin, Hao Shen, Chun-Fu Yang, Qibin Leng

**Affiliations:** 1 Key Laboratory of Molecular Virology and Immunology, Institut Pasteur of Shanghai, Shanghai Institutes for Biological Sciences, Chinese Academy of Sciences, Shanghai, China; 2 Hefei National Laboratory for Physical Sciences at Microscale, Innovation Center for Cell Biology, Institute of Immunology, School of Life Sciences, University of Science and Technology of China, Hefei, China; 3 Department of Microbiology and Immunology, Tongji University School of Medicine, Shanghai, China; 4 State Key Laboratory of Pathogen and Biosecurity, Beijing Institute of Microbiology and Epidemiology, Beijing, China; 5 Department of Microbiology, Perelman School of Medicine, University of Pennsylvania, Philadelphia, Pennsylvania, United States of America; University of Pittsburgh, UNITED STATES

## Abstract

Enterovirus 71 (EV71) is the most virulent pathogen among enteroviruses that cause hand, foot and mouth disease in children but rarely in adults. The mechanisms that determine the age-dependent susceptibility remain largely unclear. Here, we found that the paucity of invariant natural killer T (iNKT) cells together with immaturity of the immune system was related to the susceptibility of neonatal mice to EV71 infection. iNKT cells were crucial antiviral effector cells to protect young mice from EV71 infection before their adaptive immune systems were fully mature. EV71 infection led to activation of iNKT cells depending on signaling through TLR3 but not other TLRs. Surprisingly, iNKT cell activation during EV71 infection required TLR3 signaling in macrophages, but not in dendritic cells (DCs). Mechanistically, interleukin (IL)-12 and endogenous CD1d-restricted antigens were both required for full activation of iNKT cells. Furthermore, CD1d-deficiency led to dramatically increased viral loads in central nervous system and more severe disease in EV71-infected mice. Altogether, our results suggest that iNKT cells may be involved in controlling EV71 infection in children when their adaptive immune systems are not fully developed, and also imply that iNKT cells might be an intervention target for treating EV71-infected patients.

## Author Summary

Enterovirus 71 (EV71) is a major causative pathogen of hand, foot and mouth disease. EV71 infection occurs mainly in children but rarely in adults. The factors that determine the susceptibility of children to EV71 infection remain elusive. Here, we found that the paucity of invariant natural killer T (iNKT) cells in new-born mice was associated with their susceptibility to EV71 infection. Furthermore, iNKT cells played a critical role in protecting older young mice from EV71 infection before their adaptive immune systems were fully developed. Mechanistically, TLR3 signaling in macrophages, but not in dendritic cells, was essentially required for iNKT cell activation during EV71 infection. Both interleukin (IL)-12 production and endogenous lipid antigens presented by macrophages were required for full iNKT cell activation. iNKT cells tended to prevent the dissemination of EV71 into central nervous system. Taken together, our findings provide a new insight into the susceptibility of children to EV71 infection, and imply that the manipulation of iNKT cells might represent a potential therapeutic strategy for HFMD and other viral infectious diseases in children.

## Introduction

EV71 is a single-stranded, positive-sense RNA (+ssRNA) virus and belongs to the picornaviridae family. EV71 infects mainly children aged less than 5 years [1–3]. Patients with EV71 infection develop sores on the hands, feet, and sometimes buttocks and mouth, namely hand, foot and mouth disease (HFMD). Although many other enteroviruses cause HFMD in children, EV71 infection is more frequently associated with severe central-nervous-system complications in HFMD patients and thereby is a major cause of fatalities [1,4]. Thus, EV71 is considered the most virulent pathogen among the HFMD-associated enteroviruses. EV71 was first isolated from a sick child in California in 1969, and EV71 outbreaks subsequently occurred in Europe in the 1970s. Currently, HFMD is a major endemic infectious disease, with over one million cases annually in China and Southeastern Asia [3,5–7]. So far, the factors that determine the age-dependent susceptibility of children to EV71 infection remain largely unknown.

An early study by Khong et al. has shown that 2-week-old and younger immunodeficient AG129 mice, which lack type I and II interferon receptors, are susceptible to EV71 infection [8]. Their finding suggests that both type I and II interferons (IFNs) are crucial in controlling EV71 infection. Both +ssRNA and -ssRNA are produced in the life cycle of EV71. The recognition of these RNA components by TLR3, TLR7, RIG-I and MDA-5 molecules expressed by host cells potentially induces type I IFN production and limits EV71 infection. Surprisingly, the production of type I IFNs is almost absent in EV71-infected cells presumably because of inhibition by 2C and 3C proteases of the virus [9–11]. Consistently, only very low levels of type I IFNs have been detected in EV71-infected mice [8]. Furthermore, a recent study by Shih’s group has shown that mice deficient for interferon alpha receptor were not susceptible to EV71 infection. In contrast, mice deficient for interferon gamma (IFN-γ) receptor were very susceptible to EV71 infection [12]. Altogether, accumulative evidences suggest that IFN-γ, rather than type I IFNs, is likely important in limiting EV71 infection. Interestingly, it has been reported that severe HFMD patients with pulmonary edema have lower numbers of circulating leukocytes, including natural killer (NK) cells and T cells, in comparison to patients with mild disease [13]. Because technically the report has not distinguished NK cells from invariant natural killer T (iNKT) cells, it is entirely possible that iNKT cells, also IFN-γ-producing cells, may also be decreased in severe patients, and could be a link to the development of the disease.

iNKT cells are a distinct subpopulation of T cells that express an invariant αβ T cell receptor (TCR) and share a number of cell surface markers in common with NK cells. iNKT cells recognize glycolipid antigens presented by the invariant MHC class I-like molecule CD1d, which is expressed mainly on dendritic cells (DCs) and macrophages. Following lipid antigen stimulation, iNKT cells express CD40L molecule [14] and rapidly produce high levels of cytokines and chemokines [15,16]. iNKT cells are considered the first line of immune defense and are important mediators that modulate the innate and adaptive immune responses. iNKT cells are implicated in different types of viral infections, helping to control viral load or participate in disease development [17,18]. Therefore, modulating iNKT cell activation has immense clinical potential against viral infectious diseases.

In addition to microbial lipid antigens, iNKT cells recognize self-lipid antigens and are involved in the immune response to infection by microbes, which do not express glycolipids [19–25]. Paget et al. demonstrated that stimulation with TLR4, TLR7, TLR8 or TLR9 ligands renders DCs capable of promoting iNKT cell activation in a self-antigen-dependent manner. In terms of TLR9 ligand (CpG)-stimulation, iNKT cell activation fully requires type I interferon or IL-12 in dependence of the types of antigen-presenting cells in the context [24,26]. These results suggest that DNA viruses that contain CpG may trigger iNKT cell activation through the CpG-receptor TLR9. Evidently TLR9 signaling is required for iNKT cell activation in the infection by murine cytomegalovirus (MCMV), a DNA virus [27,28]. The iNKT cell activation does not require CD1d signaling but does require IL-12 production by DCs [27]. The CD1d independence and IL-12 dependence of iNKT cell activation in MCMV infection is unexpected and paradoxical to Paget’s initial observation [24] and to a later finding on microbial infections [19]. The discrepancy and the opposite role of iNKT cells [17,18] indicate that the requirement of iNKT cell activation may vary in different viral infections.

In this study, we found that lack of iNKT cells in neonatal mice was associated with their vulnerability to EV71 infection. In addition, iNKT cells played a crucial protective role in EV71-infected young mice aged less than 3 weeks when their adaptive immune systems were not fully developed. The protective immunity mediated by iNKT cells was dependent on TLR3-signaling in macrophages and the production of IL-12 and endogenous lipid ligands by macrophages. Our findings provide a new insight into the susceptibility of children to infectious diseases caused by RNA viruses, and may have implications for potential immune intervention in these diseases.

## Results

### Immaturity of the immune system is related to the susceptibility of young mice to EV71 infection

**Figure 1:**
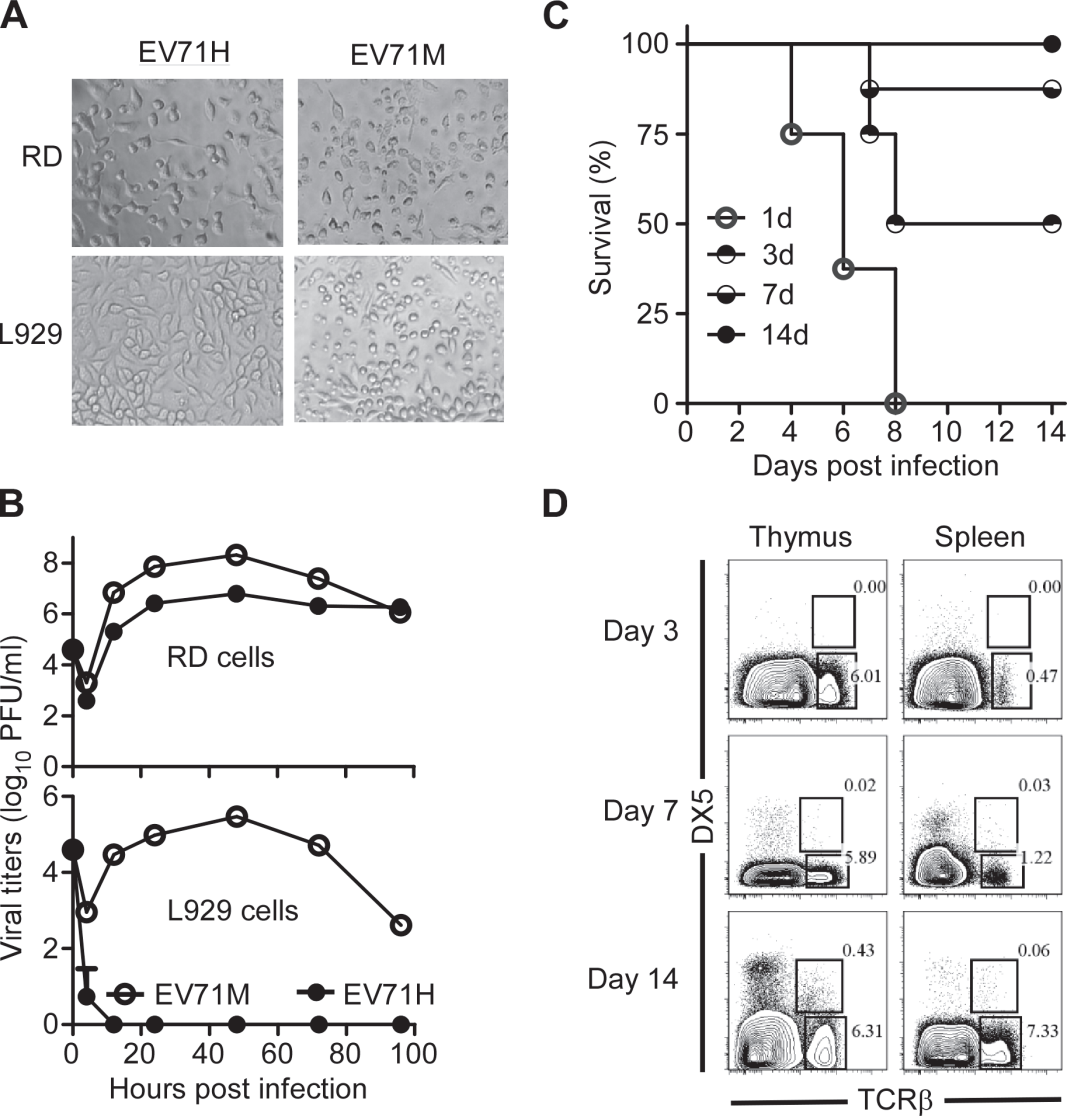
Age-dependent susceptibility of suckling mice to EV71 infection correlates with the immaturity of their immune system. (A) Cytopathic effects in human RD cells and murine L929 cells after infected with the parental human isolate EV71H and mouse-adaptive EV71M. (B) Replication kinetics of EV71H and EV71M in RD and L929 cells. (C) The survival rates of 1-, 3-, 7-, and 14-day-old ICR mice (n = 8, per group) after being infected with 2×10^6^ PFU of EV71M. (D) The proportions of NKT and T cells in the thymus or spleen of young mice aged 3, 7 and 14 days. Splenocytes were stained with TCRβ, DX5 and DAPI. TCRβ versus DX5 profiles are shown for live cells (n = 4–8). Data are representative of three (A, B, C) or two (D) independent experiments.

To obtain a mouse-adaptive EV71 (EV71M), we inoculated a clinical EV71 isolate (EV71H) into the brains of one-day-old ICR mice 5 times and then passaged the virus in L929 cells. EV71M still replicated as efficiently as the parental isolate in RD cells (Fig. 1A). In contrast to the parental EV71H virus, EV71M also replicated efficiently (increased replication up to 100-fold) in murine L929 cells 48 hours after infection (Fig. 1B). This result suggests that the mouse-adaptive EV71M gained the capability to infect murine cells.

We infected 1-, 3-, 7- or 14-day-old ICR mice with 2×10^6^ PFU of EV71M. All the one-day-old and 50% of the 3-day-old neonates developed hind limb weakness or paralysis and showed signs of encephalitis manifested by hunched posture, lethargy, and ataxia. None of the mice that developed paralysis survived after 9 days post infection. All 7-day-old mice developed at least one of the above symptoms after 6 days post infection, but their death rates dropped to 15%. All 14-day-old mice survived without any symptoms after the infection (Fig. 1C). In all, the susceptibility of EV71 infection in young mice appeared to be age-dependent.

The age-dependent susceptibility of disease development made us wonder whether the immaturity of the immune system is related to the vulnerability of neonatal mice to EV71 infection. The percentage and numbers of T, NK and NKT cells within the total lymphocyte population in the thymus and spleen of naive mice were therefore determined by flow cytometry on days 3, 7 and 14 after birth. Similar to the previous reports using C57BL/6 mice [29], the percentages of T cells were 2–3-times less in ICR neonates on days 3 and 7 of life compared with 14-day-old mice. Similarly, the percentages of NK and NKT cells were significantly lower in the younger mice. Noticeably, NKT cells were nearly undetectable in the mice before the first week of life (Fig. 1D and S1 Fig.). Thus, the small proportion of iNKT cells tends to be related to the susceptibility of neonates to EV71 infection.

### iNKT cells are activated by EV71-infected macrophages

**Figure 2:**
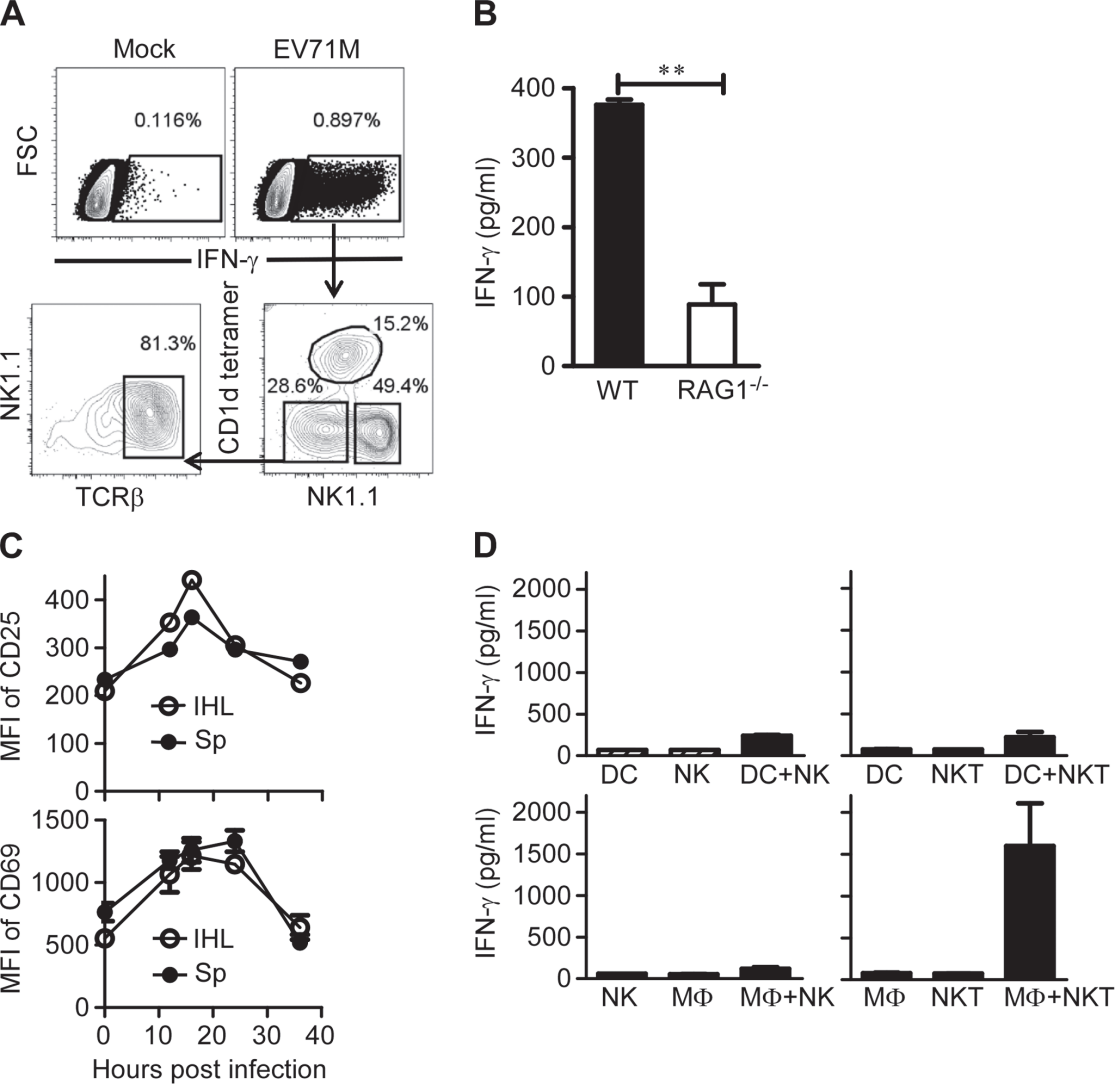
EV71 infection activates iNKT cells through triggering macrophages. (A) Increase of IFN-γ-producing cells among splenocytes after EV71M infection. Splenocytes from 6–8-week-old C57BL/6 mice (n = 3) were infected with 10 MOIs of EV71M for 18 hours and then stained with TCRβ, CD1d tetramer, NK1.1 and IFN-γ. CD1d-tetramer, TCRβ versus NK1.1 profiles are shown for IFN-γ-positive cells. (B) Splenocytes from RAG1-knockout (RAG1^-/-^) mice upon EV71M infection produced little IFN-γ. Splenocytes from WT (n = 3) and RAG1^-/-^ (n = 3) mice were cultured in the presence of EV71M for 24 hours. The supernatants were examined for IFN-γ with an ELISA assay. (C) The CD69 and CD25 expression levels of iNKT cells in the spleens (Sp) and intrahepatic leukocytes (IHL) of EV71M-infected mice (n = 3). Splenocytes of control (PBS) or EV71M-infected C57BL/6 mice were stained with TCRβ, CD1d tetramer, CD69 or CD25 and DAPI. (D) EV71M-infected macrophages, but not DCs, induced IFN-γ production by iNKT cells. Purified DCs and macrophages were cultured with EV71M for 24 hours and then co-cultured with purified NK or iNKT cells for a further 18 hours. Cytokine concentrations were determined by ELISA. Data are represented of five (A), three (mean ± SD in D) or two (B, C) independent experiments.

IFN-γ has been suggested to play a critical role in the control of EV71 infection [8,12]. To determine the cellular resources of IFN-γ production after EV71 infection, we cultured splenocytes from C57BL/6 mice with EV71M and analyzed IFN-γ production in the culture supernatants. We observed that the proportion of IFN-γ-producing cells increased 5–20 times upon EV71 infection compared with the controls (Fig. 2A and S2 Fig.). The majority of IFN-γ-producing cells were NK cells, but iNKT cells and NK1.1-negative cells also produced IFN-γ (Fig. 2A). Because NK and iNKT cells both produced IFN-γ after EV71M infection, we wondered which cells were essentially required for the IFN-γ production. RAG1-knockout mice lack NKT cells but have NK cells and other innate components. We therefore cultured RAG1-knockout splenocytes with EV71M and then examined the IFN-γ production in the cultures. We found that the IFN-γ production by the splenocytes of RAG1-knockout mice was less than one quarter of wild-type mice, suggesting that the IFN-γ production of NK cells can not be primarily triggered by EV71 infection (Fig. 2B). These observations suggest that EV71 infection primarily activates iNKT cells, and in return, presumably promotes the activation of NK cells.

To further corroborate the activation of iNKT cells by EV71 infection, we infected adult C57BL/6 mice with EV71M and examined the activation status of iNKT cells in the livers and spleens. Flow cytometry analysis revealed that iNKT cells had upregulated CD25 and CD69 expression and indeed displayed signs of activation in both tissues at 16–24 hours post-infection (Fig. 2C). Similar activation of iNKT cells was observed in the spleens of young mice (S3 Fig.).

iNKT cells can be activated by either DCs or macrophages, which are triggered by TLR ligands or microbial infections [19,24,25,30]. We next sought to determine whether EV71 infection of DCs or macrophages could activate iNKT cells. We cultured purified iNKT or NK cells together with EV71M-infected DCs or macrophages and then examined IFN-γ production in the culture supernatants. As expected, IFN-γ production was negligible in the culture supernatants of DCs, NK or iNKT cells alone. In addition, co-culture of NK cells with EV71M-infected DCs did not significantly upregulate IFN-γ production by NK cells. To our surprise, the IFN-γ production of iNKT cells was also negligible in the co-culture with EV71M-infected DCs (Fig. 2D upper panel). In contrast, the EV71M-infected macrophages dramatically induced IFN-γ production by iNKT cells (Fig. 2D lower panel). The ability of EV71M-infected macrophages to activate iNKT cells appeared not to be linked with higher level of viral replication than DCs, as the viral titers of macrophages were not significantly different from those of DCs after EV71M infection (S4 Fig.). Therefore, these results reveal that macrophages intrinsically possess the capability to specifically activate iNKT cells upon EV71 infection.

### TLR3 signaling is indispensable for iNKT cell activation in EV71 infection

**Figure 3:**
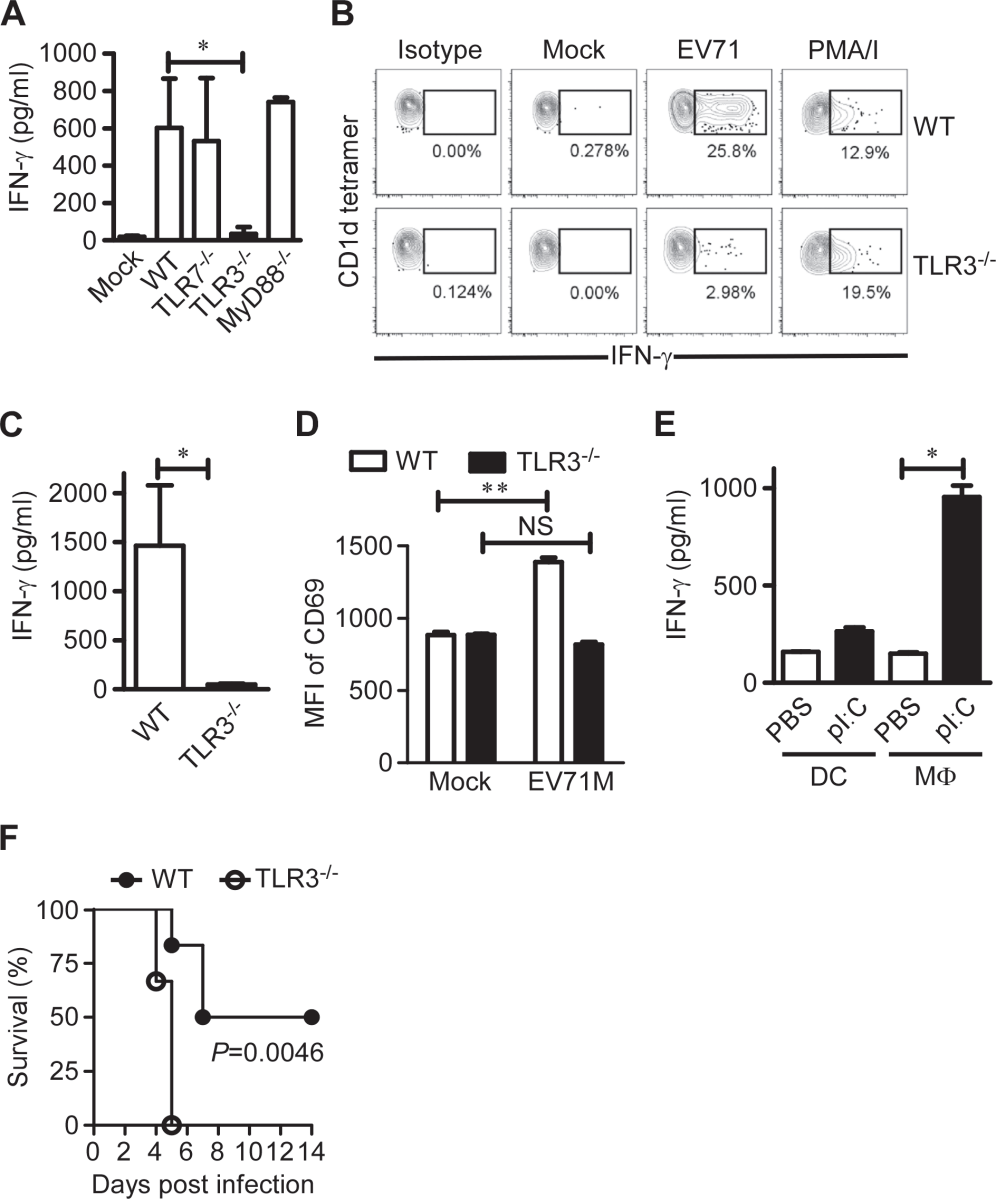
TLR3 is indispensable for iNKT cell activation in EV71 infection. (A) TLR3 signaling is required for IFN-γ production by EV71-infected splenocytes. Splenocytes from WT, TLR3^-/-^, TLR7^-/-^ and MyD88^-/-^ mice (n = 3–5) were cultured with EV71M or mock for 24 hours, and IFN-γ production was quantified by ELISA. (B) TLR3 deficiency dramatically reduced the IFN-γ production of iNKT cells. Splenocytes from CD1d-deficient or WT C57BL/6 mice (n = 3) were infected with EV71M for 24 hours and then stained with TCRβ, CD1d tetramer and IFN-γ. Splenocytes were stimulated with PMA and ionomycin (PMA/I) or mock treated (mock), which served as positive and negative controls, respectively. IFN-γ-producing cells are shown among CD1d tetramer^+^ TCR^+^-gated cells. (C) iNKT cell activation in EV71 infection is dependent on TLR3 signaling. Macrophages from WT or TLR3^-/-^ mice were cultured in the presence of EV71M for 24 hours. After extensive washing, macrophages were co-cultured for 48 hours with purified iNKT cells from WT mice, and cytokine production was quantified by ELISA. (D) TLR3 signaling is required for *in vivo* activation of iNKT cells upon EV71M infection. Six-8 week-old WT or TLR3^-/-^ mice (n = 3–6) were injected with 1×10^5^ PFU of EV71M or saline intraperitoneally. After 16 hours, splenocytes of mock (PBS) or EV71M-infected WT or TLR3^-/-^ mice were stained with TCRβ, CD1d tetramer, CD69 and DAPI. The mean fluorescent intensity (MFI) of CD69 expression on iNKT cells is shown. NS, not significant; **, *P*<0.01. (E) TLR3 triggering in macrophages activated iNKT cells. BMDCs or macrophages were cultured with medium alone or pI:C for 24 hours and then co-cultured with purified iNKT cells from WT mice for 48 hours. Cytokine production was quantified by ELISA. All results represent the mean values of cultures of 5 mice ± SD. *, *P*<0.05. (F) The survival rates of 7-day-old wild-type (WT) mice and TLR3-deficient (TLR3^-/-^) mice (n = 6, per group) after infection by 1×10^6^ PFU of EV71M. Data are representative of three (A, C, E, F) or two (B. D) independent experiments.

TLR-mediated signals and cytokines produced by antigen-presenting cells can activate iNKT cells during microbial infections in an endogenous lipid antigen-dependent manner [19,20,24]. To identify the potential pattern recognition receptor that recognizes EV71 and triggers activation of iNKT cells, we cultured splenocytes from WT mice or mice deficient for TLR3, TLR7 or MyD88 with or without EV71M and measured the production of IFN-γ in the supernatants of the cultures. TLR7- or MyD88-deficient splenocytes produced a similar level of IFN-γ as WT, TLR3-deficient splenocytes failed to produce IFN-γ in the presence of EV71M (Fig. 3A). Consistently, intracellular staining of IFN-γ revealed that iNKT cells of splenocytes from TLR3-deficient mice failed to upregulate IFN-γ expression upon EV71M challenge (Fig. 3B). Thus, these results indicate that TLR3 may be indispensable for iNKT activation in EV71M infection. To further confirm the role of TLR3 signaling in iNKT cell activation, we cultured iNKT cells purified from WT mice together with EV71M-infected macrophages from TLR3-deficient or WT mice and then examined the IFN-γ expression of iNKT cells. Our results revealed that TLR3-deficient macrophages failed to induce the production of IFN-γ by iNKT cells (Fig. 3C). It has been reported that the activation of iNKT cells by pathogens *in vitro* does not always reflect the activation requirements *in vivo* [31]. We therefore examined the expression of CD69 on iNKT cells obtained from EV71M-infected mice and WT mice. We observed that CD69 expression *in vivo* was significantly reduced on the iNKT cells from EV71-infected, TLR3-deficient mice in comparison with WT mice (Fig. 3D). Altogether, TLR3 expression in macrophages is indispensable for iNKT cell activation during EV71 infection.

Paradoxically, it has been shown that DCs stimulated with polyribo-inosinic-polyribocytidylic acid (pI:C), a TLR3 ligand, are not able to activate iNKT cells [24]. Likely macrophages harbor a specific capacity to activate iNKT cells upon TLR3 triggering. To address this possibility, we stimulated bone marrow-derived dendritic cells (BMDCs) and macrophages with pI:C and then co-cultured them with purified iNKT cells. Consistent with the previous finding [24], BMDCs stimulated with pI:C failed to upregulate IFN-γ production in iNKT cells. In contrast, macrophages stimulated with pI:C significantly upregulated the IFN-γ production of iNKT cells (Fig. 3E). This result demonstrates a cell type-specific role of TLR3 in iNKT cell activation.

To address the potential role of TLR3 in the development and/or control of disease caused by EV71 infection, WT and TLR3-deficient mice aged 7 days were infected with 1×10^6^ PFU of EV71M. At this dose of virus, half of the WT mice were paralyzed and died within 8 days post-infection, while none of the TLR3-deficient mice survived beyond 5 days post-infection (Fig. 3F). This result indicates that the TLR3-triggered immune response is indispensable for controlling EV71-caused disease in young mice.

### Endogenous CD1d ligands and IL-12 are both required for full activation of iNKT cells in EV71 infection

**Figure 4:**
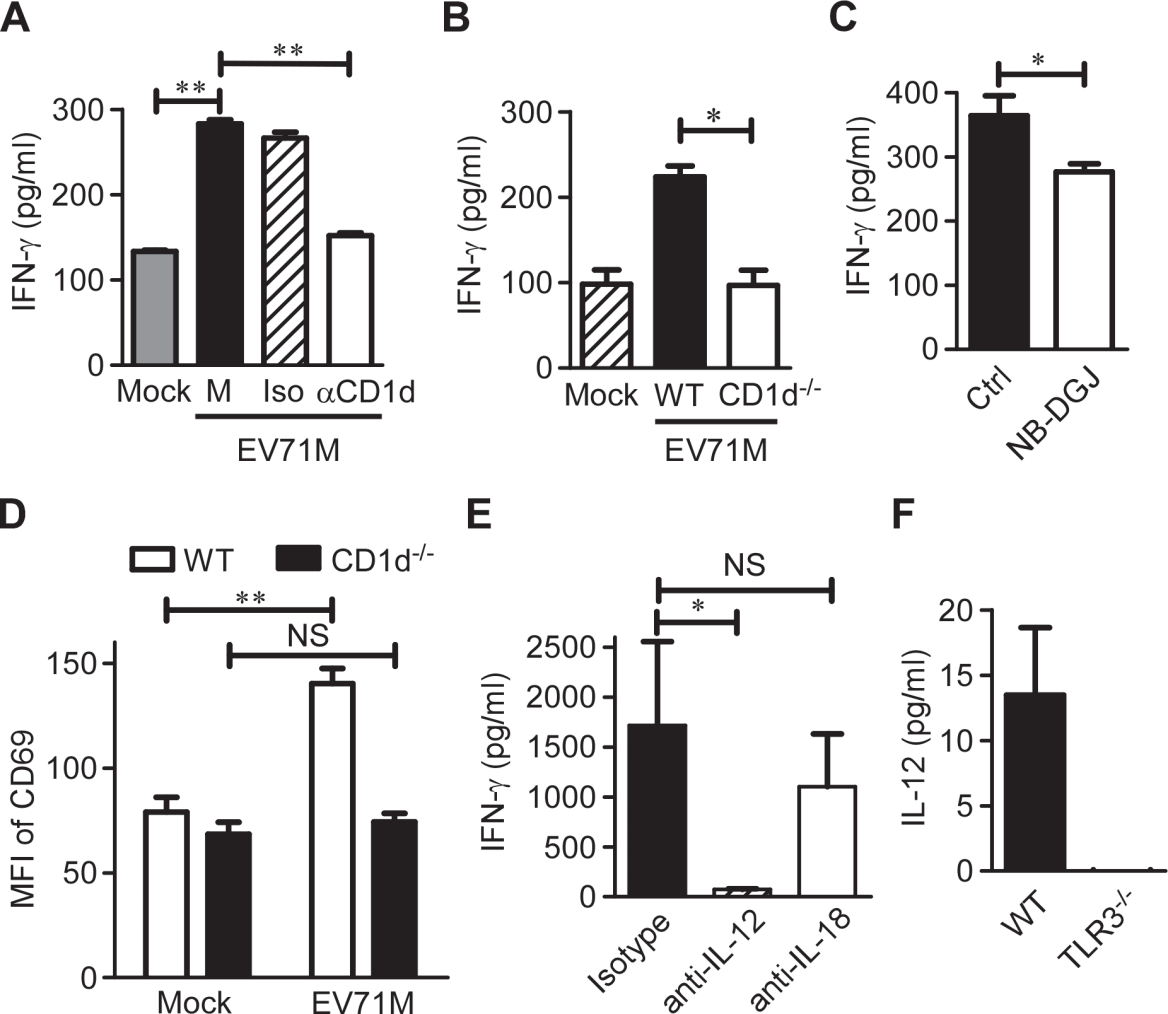
IL-12 and endogenous CD1d antigens are both required for full iNKT cell activation in EV71 infection. (A) EV71M-infected or-uninfected WT macrophages (Mock) were co-cultured with purified iNKT cells in the presence of neutralizing anti-CD1d (αCD1d), isotype control antibodies (Iso) or medium alone (M). (B) EV71M-infected or –uninfected WT or CD1d-deficient (CD1d^-/-^) macrophages were co-cultured with purified iNKT cells. (C) EV71-infected WT macrophages were co-cultured with purified iNKT cells in the presence of the lipid synthesis inhibitor NB-DGJ or medium alone. (D) Seven-day-old WT or CD1d^-/-^ neonates (n = 3–5) were adoptively transferred with 5×10^5^ purified iNKT cells or saline control intraperitoneally and infected with 2×10^5^ PFU of EV71M. After 16 hours, splenocytes of saline (mock)-treated or EV71M-infected WT or CD1d^-/-^ mice were stained with TCRβ, CD1d tetramer, CD69 and DAPI. The CD69 MFI levels on iNKT cells are shown. (E) EV71M-infected WT macrophages were co-cultured with purified iNKT cells in the presence of neutralizing anti-IL-12, anti-IL-18 or isotype control antibodies. The IFN-γ concentrations in the 24-hour culture supernatants were quantified by ELISA. (F) The IL-12 (p70) concentrations in the supernatants of WT or TLR3^-/-^ macrophages infected with 10 MOIs of EV71M. All results represent the mean ± SD. NS, not significant; *, *P*<0.05; **, *P*<0.01. Data are representative of three (A, B, C, E, F) or two (D) independent experiments.

Because EV71 per se does not encode an enzyme to synthesize lipid ligands for activating iNKT cells, we next sought to investigate whether endogenous ligands presented by CD1d molecule are required for iNKT cell activation triggered by EV71M-infected macrophages. We found that the addition of blocking antibody against CD1d molecule significantly reduced approximately 50% of the IFN-γ production of iNKT cells co-cultured with EV71M-infected macrophages (Fig. 4A). CD1d deficiency in macrophages also showed dramatically reduced IFN-γ production by iNKT cells (Fig. 4B). N-(n-butyl)-deoxy-galactonojirimycin (NB-DGJ) is an inhibitor of lactase phlorizin hydrolase, which is the first enzyme responsible for the biosynthesis of endogenous lipid antigens [24,32]. We observed that the addition of NB-DGJ also significantly inhibited the IFN-γ response of iNKT cells to EV71 infection in culture (Fig. 4C). Furthermore, we adoptively transferred purified iNKT cells into 7-day-old WT mice and CD1d^-/-^ mice and determined the CD69 expression levels of iNKT cells upon EV71M infection. We observed that CD69 expression levels were significantly upregulated on iNKT cells from WT recipient mice but not on those from CD1d^-/-^ recipient mice (Fig. 4D). Thus, iNKT cell activation in EV71 infection requires both endogenous lipid antigen synthesis and CD1d presentation.

It has been shown that IL-12 and IL-18 are involved in activating iNKT cells after TLR-mediated stimulation of DCs [19–21,23,24,33]. To investigate the contribution of cytokines that are produced by macrophages to iNKT cell activation in response to EV71 infection, we performed experiments with blocking antibodies against IL-12 and IL-18. The presence of anti-IL-12 antibody dramatically inhibited the IFN-γ production of iNKT cells co-cultured with macrophages and EV71M compared to the isotype antibody. The IL-18-blocking antibodies did not significantly affect IFN-γ production (Fig. 4E). Therefore, IL-12 production is required for iNKT cell activation.

We next investigated the effect of TLR3 signaling on IL-12 production by macrophages in response to EV71 infection. IL-12 was measured in the culture supernatant of EV71M-infected WT or TLR3-knockout macrophages. Although significant quantities of IL-12p70 were detected in the culture supernatants of EV71M-infected WT macrophages, it was nearly undetectable in the culture supernatants of EV71M-infected TLR3-knockout macrophages (Fig. 4F). Thus, IL-12 production by EV71-infected macrophages depends on TLR3 signaling.

### iNKT cells protect young mice from EV71 infection

**Figure 5:**
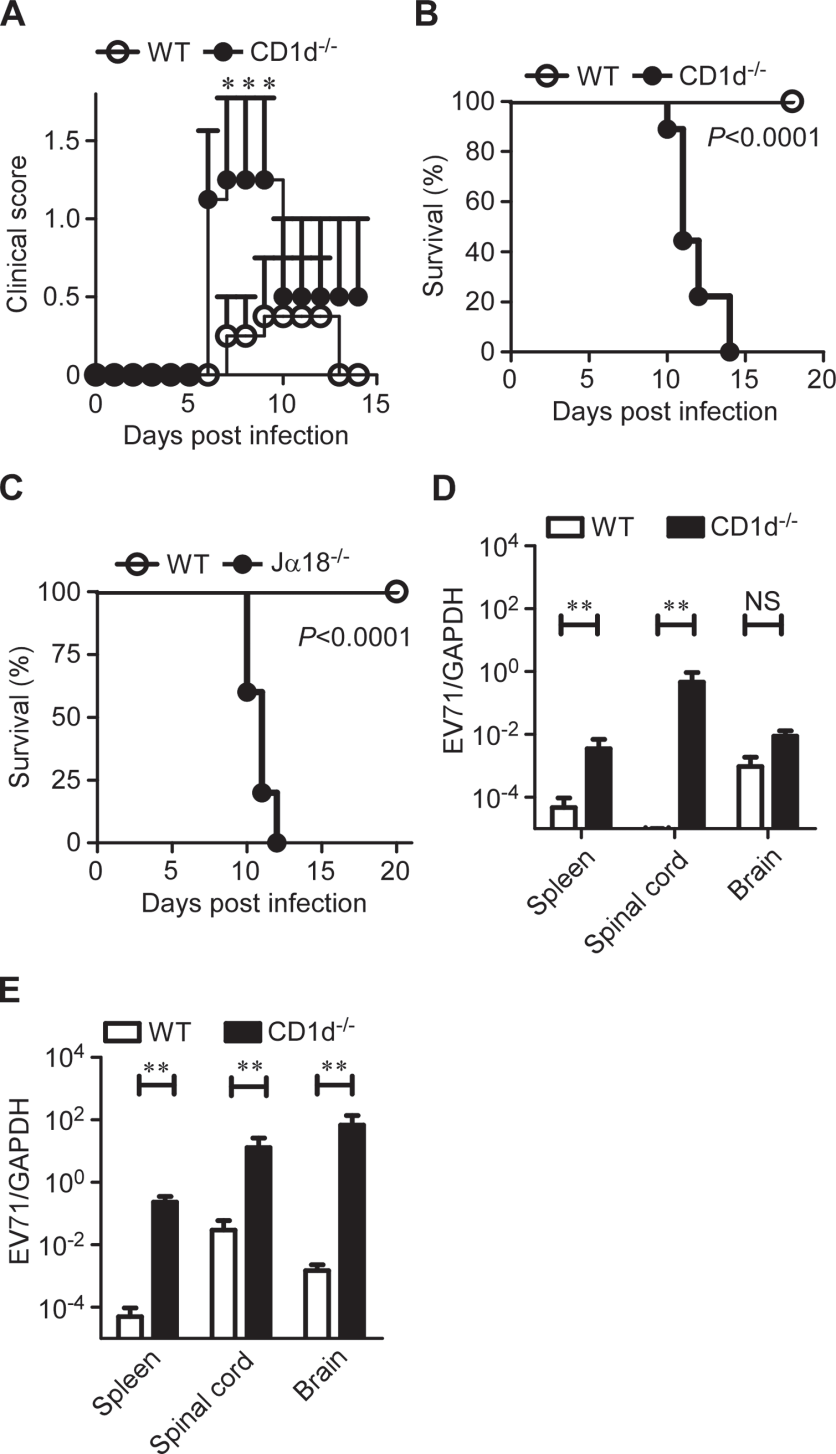
CD1d is essential for the protection of young mice from EV71 infection. Seven-day-old WT and CD1d^-/-^ mice were infected intraperitoneally with a lower dose (A, 2×10^4^ PFU) or higher dose (B, 2×10^5^ PFU) of EV71M. The clinical score (A) and survival (B, Kaplan-Meier curve) were monitored for the indicated period (n ≥ 10 per group). (C) Kaplan-Meier survival curves for 7-day-old WT and Jα18^-/-^ mice (n ≥ 5 per group) that were infected intraperitoneally with 2×10^5^ PFU of EV71M. The viral loads in each organ were examined by quantitative PCR on days 2 (D) and 4 (E) after the high dose of EV71M infection. Data are shown as the mean ± SD of three independent samples. NS, not significant; **, *P*<0.01. Data are representative of two independent experiments.

We next sought to investigate the protective or pathogenic role of iNKT cells in EV71 infection. We infected 7-day-old, CD1d-deficient or WT mice with EV71M and monitored disease development in the infected mice. At the lower dose of EV71M infection (2×10^4^ PFU), only 10% (1/10) of WT mice exhibited ruffled hair and hunchback, while 40% (6/15) CD1d-deficient mice had symptoms of limb weakness or paresis and higher average clinical scores (Fig. 5A). At the higher dose of EV71M infection (2×10^5^ PFU), all 16 CD1d-deficient mice developed limb paresis after 7 days post-infection, and none survived beyond 14 days. In contrast, none of the WT mice died of EV71M infection, although 30% (3/10) of the mice developed mild symptoms, fluffy hair, hunchback and/or limb weakness (Fig. 5B). Similarly, all the Jα18-knockout mice that lack of type I iNKT cells developed limb paresis and died within 12 days after being infected with the higher dosage of EV71M (Fig. 5C).

We further examined the virus loads in the tissues of EV71M-infected mice. We observed that the virus loads were significantly higher in the spleens and spinal cords of CD1d-deficient mice compared with WT mice on days 2 and 4 post-infection (Fig. 5D, E). Noticeably, the virus loads in the brain of CD1d-deficient mice were not significantly different from that of WT mice on day 2 post-infection but were over 1000-times higher on day 4 (Fig. 5D, E). Altogether, these results indicate iNKT cells protect young mice from EV71 infection by preventing virus spread to the neuronal system.

## Discussion

EV71 infection causes illness mainly in infants or young children. Similarly, disease development after EV71 infection only occurred in neonatal or young mice aged less than 2 weeks after adaptation [34,35]. Older human beings and mice appear to resist EV71 infection. Currently, the factors that contribute to age-dependent resistance remain elusive. Here, we found that the age-dependent susceptibility of mice to EV71 infection was reversely associated with iNKT cell development. EV71 infection triggered iNKT cell activation in a TLR3-dependent manner. Interestingly, TLR3-triggering in macrophages, rather than in BMDCs, activated iNKT cells during EV71 infection. Both IL-12 production and endogenous CD1d ligands were required for the activation of iNKT cells triggered by EV71-infected macrophages. iNKT cells appeared to prevent the spreading of EV71 into central nervous system. Furthermore, TLR3 and CD1d-deficient young mice developed more severe disease post EV71 infection. These novel findings not only help to explain the age-dependent susceptibility of host to EV71 infection but also provide initial knowledge about how infections by RNA viruses trigger activation of iNKT cells.

iNKT cells begin to develop in mice approximately 7 days after birth (Fig. 1D and S1 Fig.) [36]. We found that wild-type mice are susceptible to EV71M infection under this age. iNKT-deficient mice under 3 weeks of age are more susceptible to EV71M infection when compared with wild-type mice. It is known that the adaptive immune system begins to mature at this time [37]. Therefore, the availability of iNKT cells in early life is an important factor to control EV71 infection. In other words, iNKT cells play a critical role in protecting mice from the virus infection between the second and third weeks of their life, before the adaptive immune system becomes fully functional.

The mechanism underlying how infections of RNA viruses trigger iNKT cell activation has not been well-elucidated. Nevertheless, it has been reported that DCs stimulated by ligands of TLR7 and TLR8, pattern recognition receptors sensing RNA viruses, are capable of activating iNKT cells. Paradoxically, DCs stimulated by the dsRNA analog pI:C, TLR3 ligand, fail to activate iNKT cells [24]. Thus, one can expect that the ssRNA sensors TLR7 and TLR8 are required for iNKT cell activation, and that TLR3 signaling unlikely contributes to iNKT cell activation during RNA virus infection. In contrast to the assumption, our present study revealed that TLR3, rather than TLR7 and other TLRs, was crucial in iNKT cell activation during EV71 infection. Macrophages but not DCs were indispensable antigen-presenting cells for activation of iNKT cells.

The incapability of TLR3-mediated signaling in DCs to activate iNKT cells highlights that there is an intrinsic difference in TLR3 signaling pathways between macrophages and DCs. This merits further investigation. However, one caveat is that DCs in our and others’ experiments were *in vitro*-cultured BMDCs. Unlike *in vitro* BMDCs, the DCs in lymphoid organs and other tissues of humans and mice are heterogeneous in phenotype and functionality [38]. Thus, it is impossible to exclude some subsets of DCs that may activate iNKT cells upon RNA virus infection *in vivo*. Furthermore, fecal-oral infection is a natural route of transmission of EV71 infection. CD1d also expresses on the intestine epithelial cells and is involved in the induction of oral tolerance and protection from mucosal infections [39]. We also observed that 75% of CD1d-knockout mice upon oral infection of EV71M developed disease and had significantly higher clinical score than WT mice, as all the wild type mice survived without any palpable symptoms after the infection (S5 Fig.). Therefore, it is also possible that intestine epithelial cells also play a role in the mucosal immunity elicited by iNKT cells against EV71 infection. It is also of great interest to investigate the cell type-dependent activation of iNKT cells in EV71 infection as well as other viral infections in future.

Viruses mostly do not encode enzymes responsible for synthesizing glycolipids that bind to CD1d molecule. Our results showed that blocking CD1d-mediated antigen presentation with antibody and biosynthesis of endogenous lipid antigens in EV71-infected macrophages with an inhibitor significantly decreased the activation of iNKT cells. In addition, CD1d-deficient macrophages post EV71 infection had significantly reduced ability to activate iNKT cells compared to WT macrophages. Furthermore, adoptive transfer of purified iNKT cells also slightly but significantly prolonged the survival of EV71M-infected 3-day-old WT mice, but this protective effect did not occur in CD1d-deficient recipients (S6 Fig.). Similarly, adoptive transfer experiment also suggested that activation of iNKT cells *in vivo* in EV71M infection also requires CD1d molecule (Fig. 4D). These results all suggested that full iNKT cell activation during EV71 infection required CD1d molecule and its presentation of endogenous lipid antigens. This is in contrast to the findings showing that iNKT cell activation triggered by the infection of MCMV is not dependent on CD1d molecule [27,28]. MCMV encodes a CD1d-like protein, m157, that can directly bind to the Ly49H molecule on NK cells and activate the NK cells [40,41]. Ly49H is still expressed on NK1.1-positive cells in Nfil3-deficient mice that have no NK cells but still have iNKT cells [42], suggesting that iNKT cells may potentially express Ly49H. The potential Ly49H-m157 interaction may thus contribute to the CD1d independence of iNKT cell activation. Altogether, the discrepancy in the CD1d requirement in viral infections may support a notion that different viruses may trigger distinct pathways to activate iNKT cells.

Overall, we showed that iNKT cells are crucial antiviral effectors against EV71 infection before the adaptive immune system becomes fully functional in mice. Our result of human peripheral blood mononuclear cells (PBMCs) demonstrated that human iNKT cells can also be activated by EV71 infection (S7 Fig.). Children under the age of 5 years are more frequently susceptible to EV71 infection as well as other RNA virus infections [5,6], including infections by influenza A virus, respiratory syncytial virus, human metapneumovirus, coronaviruses and rhinovirus than adults [43–45]. Thus, our results encourage further investigations on the role of iNKT cells in childhood diseases caused by these viruses and exploring therapeutic strategies against these viral infections by manipulating iNKT cells.

## Materials and Methods

### Ethics statement on human subjects

Use of human PBMCs from healthy donors was approved by the Institutional Ethics Committee of the Institut Pasteur of Shanghai (Permit Number: IPS-2012001).

### Ethics statement on animal subjects

All animal experiments were performed in strict accordance with the regulations in the Guide for the Care and Use of Laboratory Animals issued by the Ministry of Science and Technology of the People's Republic of China. All efforts were made to minimize suffering. The protocol was approved by the Institutional Animal Care and Use Committee of the Institut Pasteur of Shanghai (Permit Number: A2011006).

### Mice

C57BL/6 and ICR mice were obtained from Shanghai Laboratory Animal Center (SLAC). TLR3^-/-^ mice were kindly provided by Dr. Richard Flavell and have been backcrossed to C57BL/6 background for more than eight generations. RAG1^-/-^ mice and MyD88^-/-^ mice with the C57BL/6 background were provided by the Model Animal Research Center of Nanjing University. B6.129S1-Tlr7^tm1Flv^/J (TLR7^-/-^) mice in C57BL/6 background were purchased from the Jackson Laboratory. Jα18^-/-^ mice in C57BL/6 background were originally obtained from M. Taniguchi (RIKEN Research Center, [46]). Vα14-Jα18 transgenic mice in C57BL/6 background were generated as previously described [47]. All mice were kept under specific pathogen-free (SPF) conditions in the SLAC. Infections were performed in containment isolators under SPF conditions. Animals were infected intraperitoneally (i.p.) with the indicated dose of EV71M in 50 μl RPMI-1640 medium. The clinical scores of the mice after EV71M infection were as follows: 0, healthy; 1, ruffled hair and hunchback; 2, limb weakness; 3, paralysis in 1 limb; 4, paralysis in both limbs; and 5, death.

### Reagents and antibodies

pI:C, PMA, ionomycin and bredfeldin A were purchased from Sigma-Aldrich. Anti-CD4, anti-CD3, anti-TCRβ, anti-CD19, anti-CD25, anti-CD69, anti-DX5, anti-CD11b, anti-CD11c, anti-NK1.1, anti-IFN-γ and isotype-matched control antibodies were purchased from eBioscience. Blocking antibodies against CD1d, IL-12 and IL-18 were also purchased from eBioscience.

### Cells and viruses

RD cells were cultured in Dulbecco's modified Eagle’s medium (HyClone) supplemented with 10% fetal calf serum (FCS). Mouse fibroblast L929 cells were grown in RPMI-1640 medium (HyClone) with 10% FCS. The clinical strain EV71H was originally isolated from a child who suffered EV71 encephalomyelitis in 2008 [48].

The mouse-adapted virus strain (EV71M) was generated following a previously described method [49]. In brief, 1-day-old ICR mice (n = 8) were intracerebrally inoculated with 1×10^4^ PFU of the parental strain. Brains of the infected mice were collected and pooled 4 days post-infection, and homogenates of the brains were prepared in HBSS at a ratio of 10% weight/volume. The virus in the homogenate was cultured in L929 cells. The stock virus was harvested at the fifth passage and titrated in RD cells. The final virus stock was 4×10^8^ PFU/ml. All experiments with EV71 virus were performed in a biosafety level-2 laboratory at the Institut Pasteur of Shanghai.

RD and L929 cell monolayers in 24-well plates (2–3×10^5^ cells) were infected with the parental strain EV71H or EV71M at a multiplicity of infection (MOI) of 0.1 for 1 hour at 37°C. The cells were washed twice with PBS and then cultured in DMEM containing 2% FCS. Samples were harvested at various indicated time points. At each time point, the supernatant was stored in aliquots at -80°C and later titrated by a plaque assay using RD cell monolayers in 24-well plates.

### BMDCs and peritoneal macrophages

BMDCs were generated as described previously [50]. Briefly, bone marrow was harvested from tibias and femurs of different mice. After lysis of red blood cells, bone marrow cells were cultured for 6 days in RPMI-1640 medium supplemented with 10% FCS and supernatant from GM-CSF-expressing J5 cells, which were kindly provided originally by Dr. Herman Eisen. CD11c^+^ cells were further purified by positive selection using MACS separation columns (Miltenyi Biotec) according to the manufacturer’s instructions. The purified cells were above 96% CD11c positive.

The murine peritoneal macrophages were harvested from 6–8-week-old C57BL/6 mice after the i.p. injection of 1 ml of 4% thioglycollate medium (Sigma-Aldrich) as described previously [51]. Before EV71 infection, the macrophages were cultured overnight in serum-free RPMI-1640 with antibiotics to minimize the influence of FBS.

### Purification of iNKT cells

For iNKT cell adoptive transfer experiments, a single-cell suspension was prepared from Vα14-Jα18 transgenic mouse spleens. iNKT cells were prepared using the modified protocol of Pei [52]. Briefly, freshly isolated spleens were mashed and passed through a 40-μm nylon strainer to give a single-cell suspension. Cells from three spleens were pooled, and RBCs were removed by lysis. The splenocytes were stained with CD1d-tetramer or antibodies against NK1.1 and TCRβ and then sorted using a FACS Aria II cell sorter (BD Biosciences) (≥97% pure).

### Infection of DCs and macrophages and co-culture with iNKT cells

Macrophages or DCs (2×10^5^ cells/well in a U-bottomed, 96-well plate) were incubated with EV71 (MOI = 10) or TLR agonists for 24 hours, extensively washed with PBS, and cultured for 24 hours with spleen iNKT cells (1×10^5^ /well) in U-bottomed, 96-well plates containing RPMI 1640 medium supplemented with 10% FBS. In some cases, neutralizing or control antibodies were added during the co-culture. The IFN-γ concentrations in the co-culture supernatants were measured by ELISA (eBioscience). To inhibit GSL synthesis, macrophages were treated with 100 μg/ml NB-DGJ for 24 hours prior to the iNKT cell assays. NB-DGJ was also present during the stimulation experiments at 100 μg/ml.

### Isolation and cultivation of human PBMCs

Human PBMCs were isolated from whole blood via Ficoll-Hypaque density gradient centrifugation and subsequently washed with RPMI-1640 before further use. For examining iNKT cell activation, EV71-infected or mock-treated PBMCs were resuspended in RPMI-1640 supplemented with 10% FCS and seeded at 5×10^6^ cells/ml in 24-well plates for 16 hours.

### Flow cytometry

Cells were washed and blocked in staining buffer (PBS, 0.3% BSA and 0.1% sodium azide) containing anti-CD16/CD32 for 10 minutes at 4°C then stained with fluorophore-conjugated antibodies. After washing twice with staining buffer, data were collected on an LSRII flow cytometer (BD Biosciences). For intracellular staining, 2.5 μg/ml BFA was added during the last 4 hours of stimulation to block the secretion of cytokines. The cells were washed and stained for cell-surface markers. After fixation and permeabilization with Cytofix/Cytoperm Kit (BD pharmingen) according to the manufacturer’s protocol, the cells were stained with FITC-anti-mouse IFN-γ or isotype control and analyzed with an LSR II flow cytometer. The data were analyzed using FlowJo software (Tree Star).

### RNA extraction, cDNA synthesis and real-time PCR

Total RNA was isolated from EV71-infected or un-infected cells or tissues with TRIzol reagent (Invitrogen), and cDNAs were synthesized from 1 μg of total RNA with random hexamer primers and Superscript reverse transcriptase (TAKARA Biotechnology Co., Ltd) according to standard procedures. cDNAs were used as templates for PCR amplification using the SYBR Green PCR Master Mix (Takara Biotechnology Co., Ltd) and the ABI 7900HT Fast Real-Time PCR System (Applied Biosystems, Foster City, CA). Primers for EV71 and GAPDH were previously described by Xu et al. and Paget et al., respectively [53] [24].

### Cytokine analysis

Samples were harvested and stored at -80°C before analysis. The IFN-γ and IL-12 levels were quantified using ELISA Ready-SET-Go (eBioscience).

### Statistical analyses

Statistical analyses for continuous data were performed with Prism5 for Windows software (Prism Graph-Pad Software Inc) using two-tailed Student’s *t*-tests. *P* < 0.05 was considered significant. Statistical differences in mouse survival were determined by Gehan-Breslow-Wilcoxon Test analysis. Graphs were produced and statistical analyses were performed using GraphPad Prism.

## Supporting Information

S1 Fig The proportions of iNKT and T cells in the thymus or spleen of young mice aged 3, 7 and 14 days. Thymus cells and splenocytes from ICR (A) or C57BL/6 (B) mice were stained with TCRβ, CD1d tetramer and DAPI. TCRβ versus CD1d tetramer profiles are shown for live cells. Data are representative of two independent experiments (n = 3–5 mice per group).
(TIF)

S2 Fig EV71M infection induces IFN-γ expression of iNKT cells from suckling mice *in vitro*. Splenocytes from 7 or 14-day-old C57BL/6 mice were infected with EV71M for 24 hours and then stained with TCRβ, CD1d tetramer and IFN-γ. Splenocytes that were stimulated with PMA and ionomycin (PMA/I) or mock treated (mock) served as positive and negative controls, respectively. IFN-γ-producing cells are shown among CD1d tetramer^+^TCRβ^+^-gated cells. Splenocytes from more than three mice were pooled and infected in triplicate. Data are representative of two independent experiments.
(TIF)

S3 Fig EV71M infection activates iNKT cells in suckling mice. Two-week-old C57BL/6 mice (n = 6–10) were infected with 2×10^5^ PFU of EV71M. Splenocytes of EV71M-infected mice were stained with TCRβ, CD1d tetramer, CD25, CD69 and DAPI. The CD25 and CD69 expression levels of CD1d tetramer^+^TCRβ^+^-gated cells are shown for live cells. Data are representative of two independent experiments.
(TIF)

S4 Fig One-step growth curves of EV71M infection in immature DCs and macrophages. Immature DCs (iDCs) and macrophages (MΦ) were inoculated at an MOI of 10 and the titers of cell-associated virus were determined at various time points post-infection on RD cells. Each curve is the average of two independent experiments.
(TIF)

S5 Fig CD1d is essential for the protection of young mice from EV71 infection. Seven-day-old WT and CD1d^-/-^ mice were inoculated with a high dose (2×10^5^ PFU) of EV71M using orogastric gavage. The clinical scores were monitored for the indicated period (n ≥ 4 per group).
(TIF)

S6 Fig CD1d is essential for the protection elicited by iNKT cells from EV71 infection. Three-day-old WT (circle) or CD1d^-/-^ (triangle) neonates were adoptively transferred with 2×10^5^ purified iNKT cells (filled) or vehicle control (hollow) intraperitoneally and infected with 2×10^5^ PFU of EV71M. Survival was monitored for the indicated period (n ≥ 3 per group). Kaplan-Meier curves were plotted using GraphPad Prism and difference between the WT mice transferred with iNKT cells group and CD1d^-/-^ transferred with iNKT cells group or PBS-injected groups was statistically significant as determined by Gehan-Breslow-Wilcoxon Test analysis (*P* < 0.05).
(TIF)

S7 Fig EV71 infection activates human iNKT cells. PBMCs from 4 healthy adult subjects (Sub) were cultured with 4 MOIs of EV71M or mock for 16 hours, and stained with CD3, CD1d tetramer, CD69 and DAPI. The frequencies of iNKT cells among mononuclear cells were shown (upper panel). CD69 Expression profiles of CD1d tetramer^+^CD3^+^-gated cells were evaluated by flow cytometry for each infected samples (black line) relative to mock controls (gray) (lower panel).
(TIF)
