# Supplementary figures and images for: TLR3 Signaling in Macrophages Is Indispensable for the Protective Immunity of Invariant Natural Killer T Cells against Enterovirus 71 Infection
Source: PLoS Pathog. 2015 Jan 23;11(1):e1004613. doi: 10.1371/journal.ppat.1004613 (PMC4304831; doi:10.1371/journal.ppat.1004613)

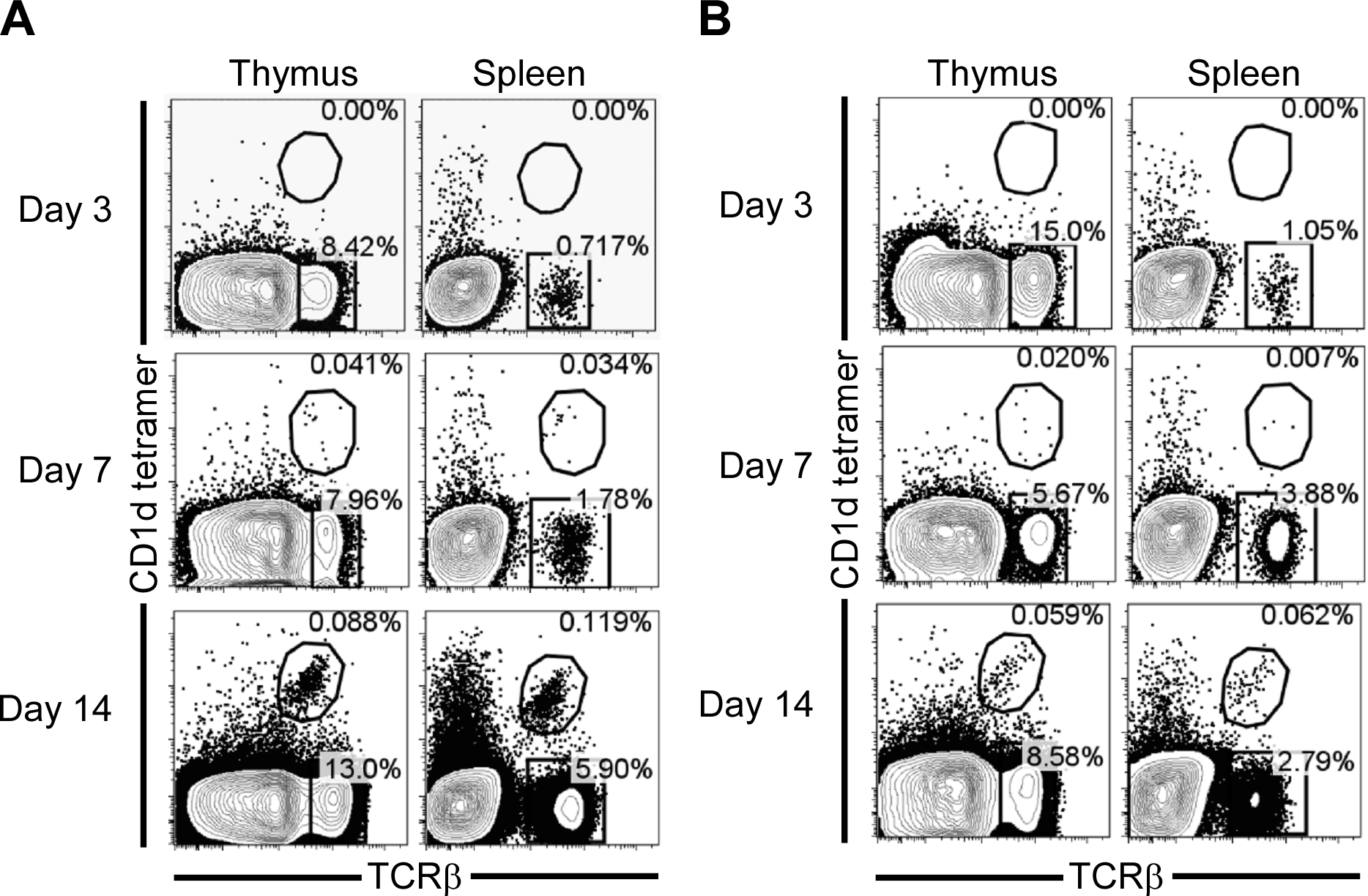

Supplement: S1 Fig — Thymus cells and splenocytes from ICR (A) or C57BL/6 (B) mice were stained with TCRβ, CD1d tetramer and DAPI. TCRβ versus CD1d tetramer profiles are shown for live cells. Data are representative of two independent experiments (n = 3–5 mice per group). (TIF) [file ppat.1004613.s001.tif]

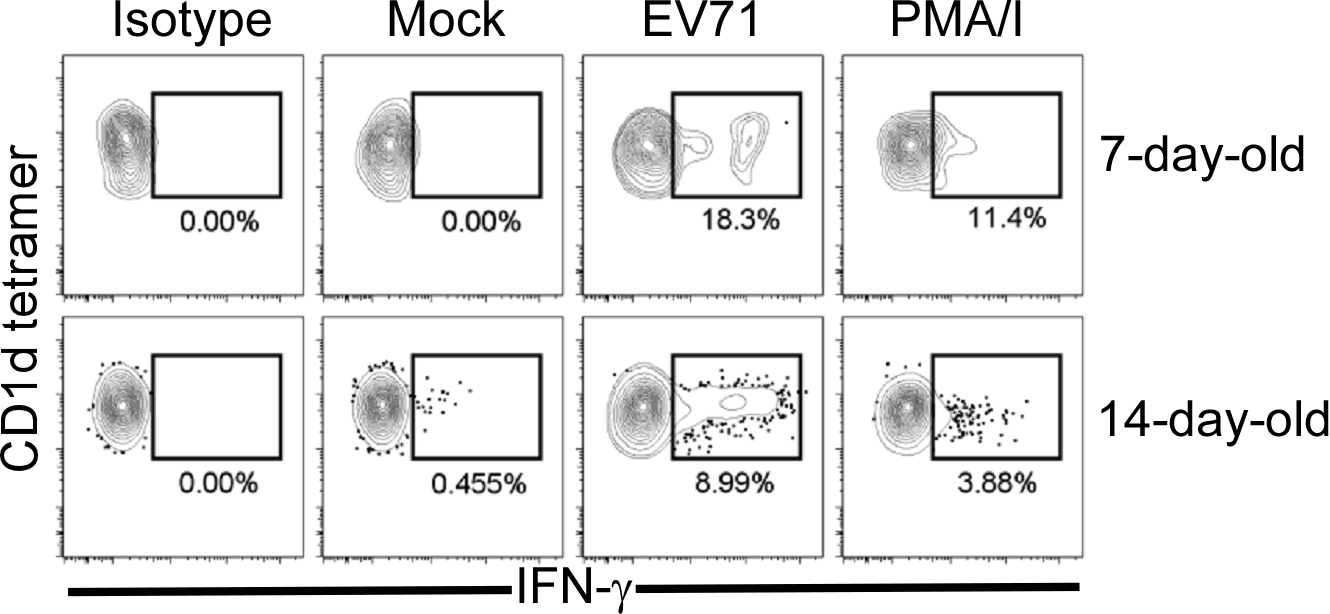

Supplement: S2 Fig — Splenocytes from 7 or 14-day-old C57BL/6 mice were infected with EV71M for 24 hours and then stained with TCRβ, CD1d tetramer and IFN-γ. Splenocytes that were stimulated with PMA and ionomycin (PMA/I) or mock treated (mock) served as positive and negative controls, respectively. IFN-γ-producing cells are shown among CD1d tetramer+TCRβ+-gated cells. Splenocytes from more than three mice were pooled and infected in triplicate. Data are representative of two independent experiments. (TIF) [file ppat.1004613.s002.tif]

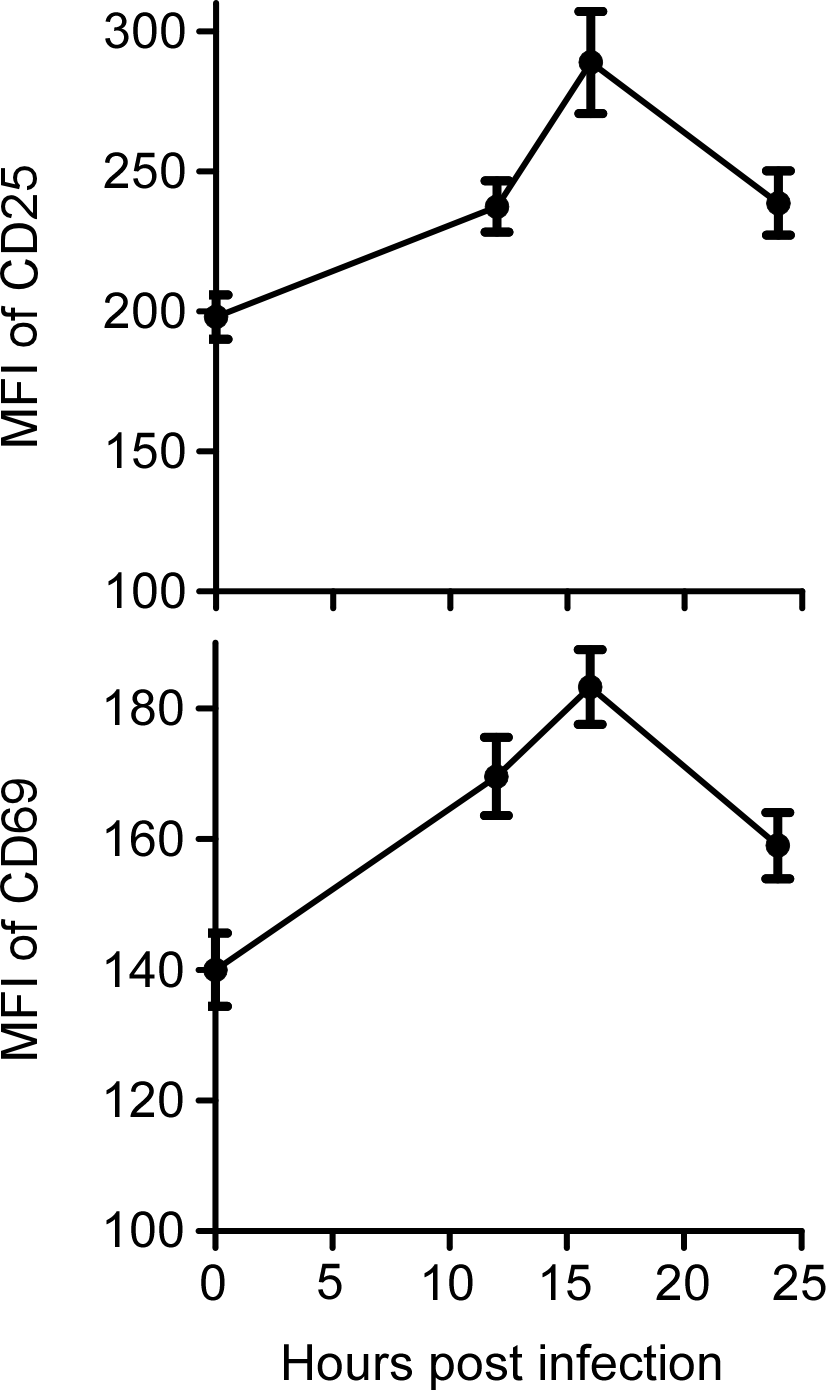

Supplement: S3 Fig — Two-week-old C57BL/6 mice (n = 6–10) were infected with 2×10^5^ PFU of EV71M. Splenocytes of EV71M-infected mice were stained with TCRβ, CD1d tetramer, CD25, CD69 and DAPI. The CD25 and CD69 expression levels of CD1d tetramer+TCRβ+-gated cells are shown for live cells. Data are representative of two independent experiments. (TIF) [file ppat.1004613.s003.tif]

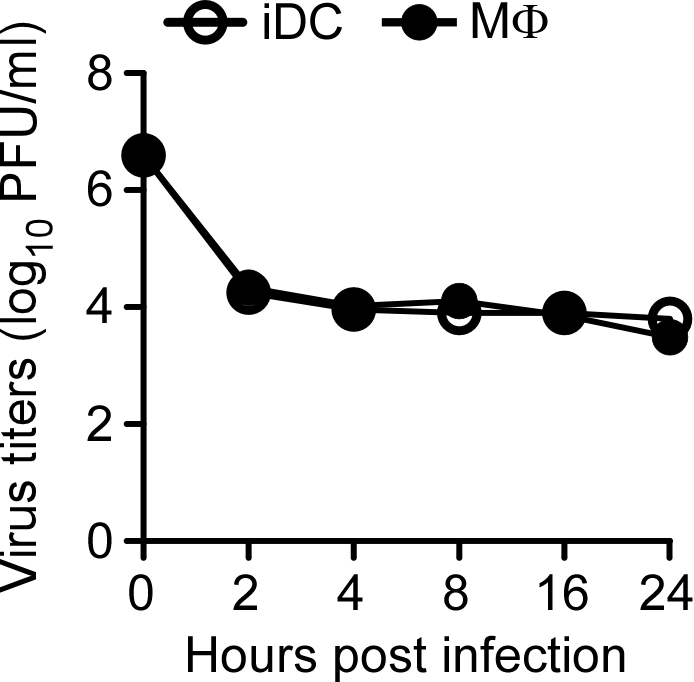

Supplement: S4 Fig — Immature DCs (iDCs) and macrophages (MΦ) were inoculated at an MOI of 10 and the titers of cell-associated virus were determined at various time points post-infection on RD cells. Each curve is the average of two independent experiments. (TIF) [file ppat.1004613.s004.tif]

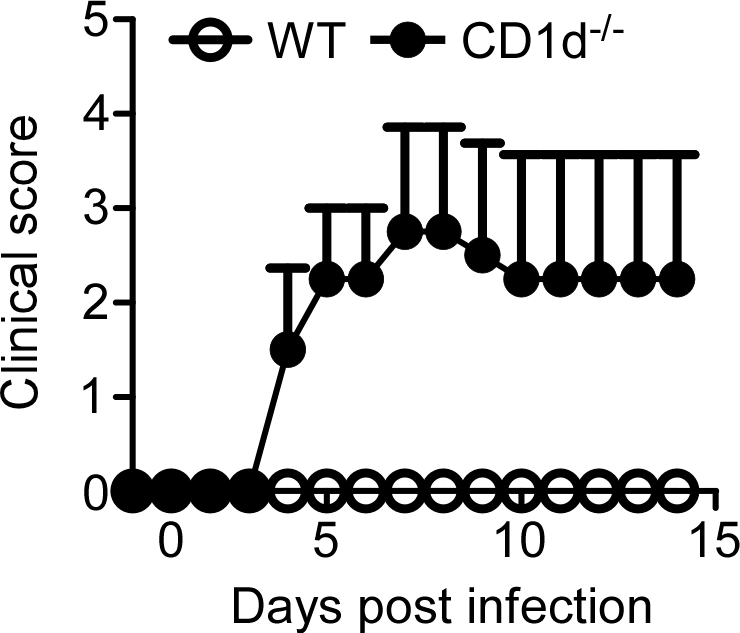

Supplement: S5 Fig — Seven-day-old WT and CD1d-/- mice were inoculated with a high dose (2×10^5^ PFU) of EV71M using orogastric gavage. The clinical scores were monitored for the indicated period (n ≥ 4 per group). (TIF) [file ppat.1004613.s005.tif]

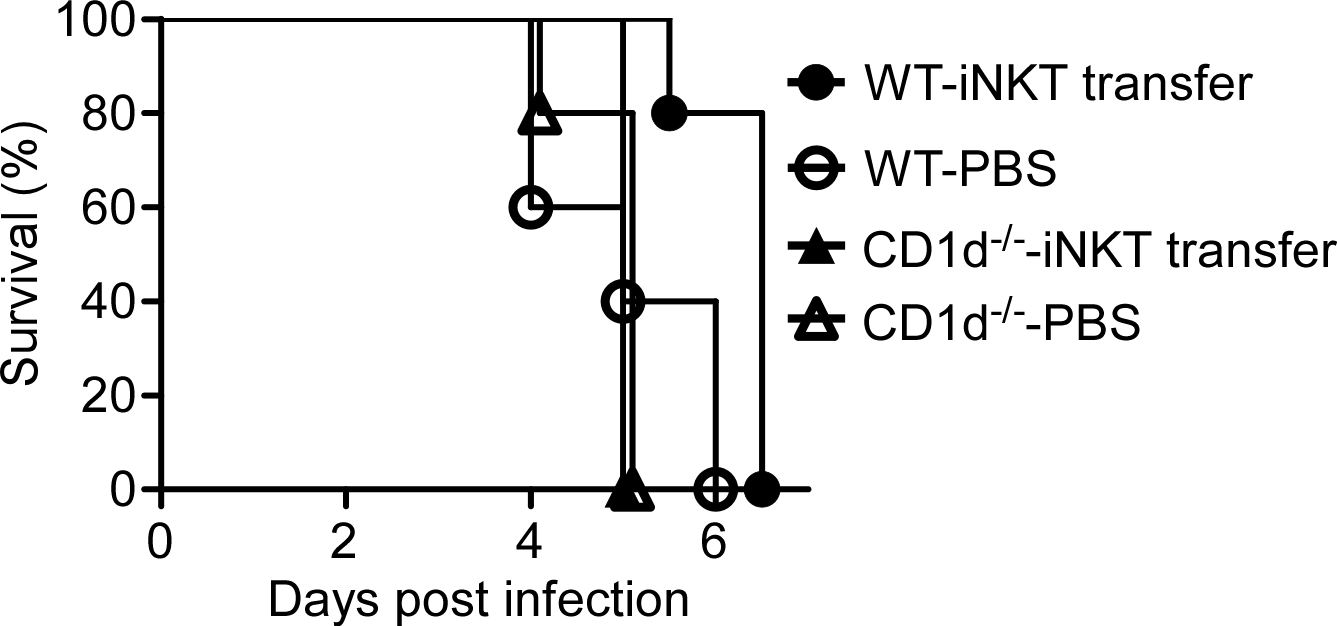

Supplement: S6 Fig — Three-day-old WT (circle) or CD1d-/- (triangle) neonates were adoptively transferred with 2×10^5^ purified iNKT cells (filled) or vehicle control (hollow) intraperitoneally and infected with 2×10^5^ PFU of EV71M. Survival was monitored for the indicated period (n ≥ 3 per group). Kaplan-Meier curves were plotted using GraphPad Prism and difference between the WT mice transferred with iNKT cells group and CD1d-/- transferred with iNKT cells group or PBS-injected groups was statistically significant as determined by Gehan-Breslow-Wilcoxon Test analysis (P < 0.05). (TIF) [file ppat.1004613.s006.tif]

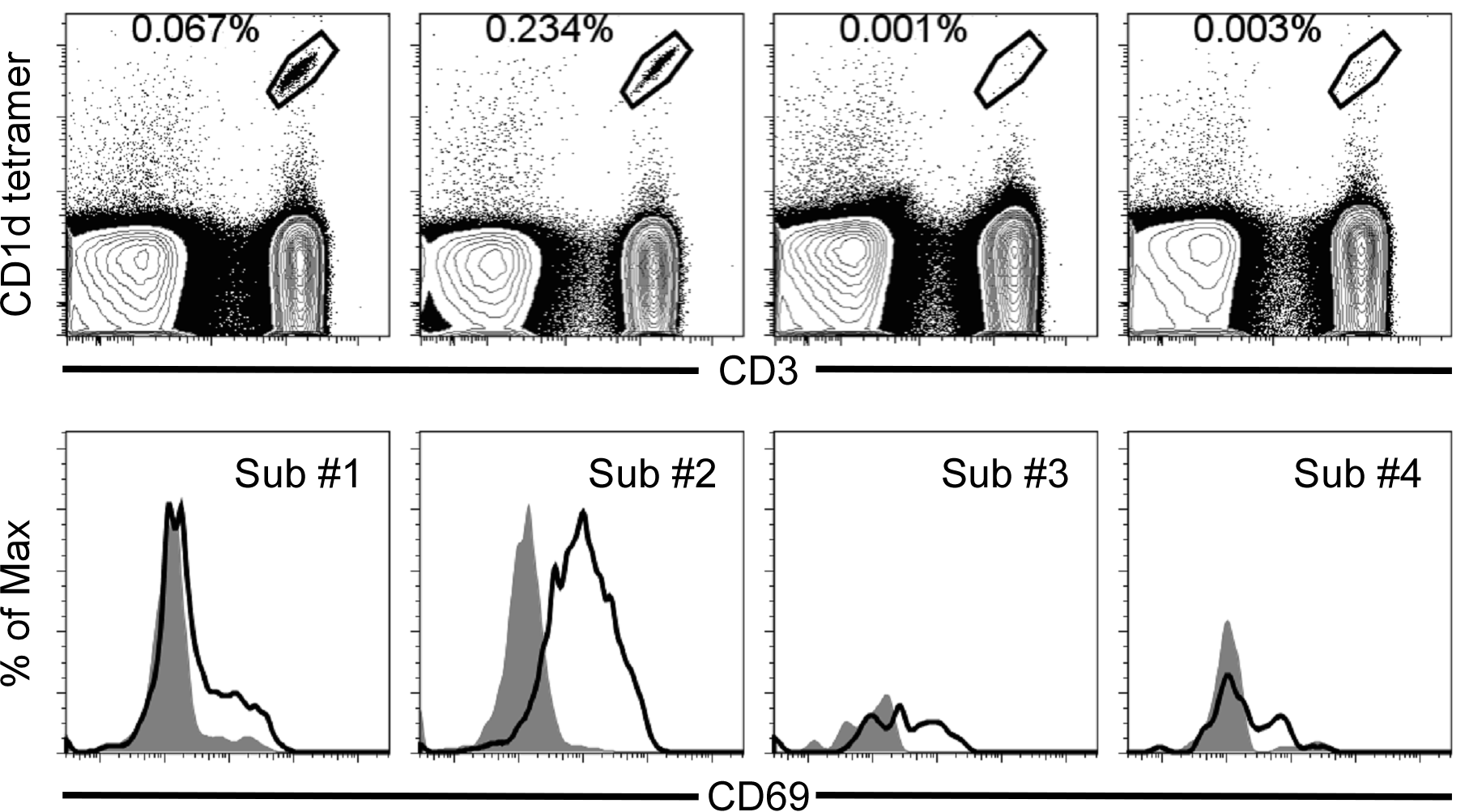

Supplement: S7 Fig — PBMCs from 4 healthy adult subjects (Sub) were cultured with 4 MOIs of EV71M or mock for 16 hours, and stained with CD3, CD1d tetramer, CD69 and DAPI. The frequencies of iNKT cells among mononuclear cells were shown (upper panel). CD69 Expression profiles of CD1d tetramer+CD3+-gated cells were evaluated by flow cytometry for each infected samples (black line) relative to mock controls (gray) (lower panel). (TIF) [file ppat.1004613.s007.tif]
